# Outbreak of severe myocarditis in England: Havoc by a harmless virus

**DOI:** 10.1002/hsr2.1541

**Published:** 2023-08-31

**Authors:** Heeba Anis, Akbar Basha Shaik, Angad Tiwari, Abel Alemayehu, Abubakar Nazir, Linda Atulinda, Magda Wojtara, Olivier Uwishema

**Affiliations:** ^1^ Department of Research Oli Health Magazine Organization, Research, and Education Kigali Rwanda; ^2^ Department of Medicine Deccan College of Medical Sciences Hyderabad Telangana India; ^3^ Department of Medicine Maharani Laxmi Bai Medical College Jhansi Uttar Pradesh India; ^4^ Department of Medicine, College of Health Science Addis Ababa University Addis Ababa Ethiopia; ^5^ Department of Medicine Makerere University Kampala Uganda; ^6^ Department of Medicine University of Michigan Medical School Ann Arbor Michigan USA; ^7^ Department of Medicine Clinton Global Initiative University New York USA; ^8^ Department of Medicine Karadeniz Technical University Trabzon Turkey

**Keywords:** critical care, enterovirus, myocarditis, public health

## Abstract

**Background:**

The World Health Organization (WHO) has been alerted to a concerning upsurge of severe myocarditis, an inflammatory heart condition, in neonates within Wales and South West England. The myocarditis cases are being intricately associated with enterovirus infection, belonging to the Picornaviridae family. The concerned pathogen poses a significant global disease burden, with an estimated 10 to 15 million symptomatic cases occurring annually in the United States alone. Neonates are particularly vulnerable with children under the age of one accounting for approximately 40% of enterovirus infections.

**Material and Methods:**

A comprehensive literature search was conducted using various databases including ClinicalTrials, Google Scholar, PubMed, ScienceDirect, MEDLINE, and Ovid Resources. The search strategy included utilizing keywords such as “myocarditis,” “Randomized controlled trials (RCTs). Only articles written in English were considered, and selection criteria included relevance to the research objectives, reasonable sample sizes, and robust methodology. In addition to the identified articles, meta‐analyses, animal models and studies, and references from the selected articles were also examined to ensure a comprehensive review of the literature.

**Results:**

Ten hospitalized neonates, reported in the United Kingdom (UK), with positive PCR tests were reported to have myocarditis, predominantly caused by coxsackieviruses. The current situation in the region has brought global attention. With this study, we hope to draw attention to the critical aspects of the illness and, more crucially, the present strategies required to control the disease outbreak in England.

**Discussion:**

Current European Society of Cardiology (ESC) recommendations for the treatment of acute heart failure apply. Emerging research supports the use of immunosuppressive medication in some circumstances. Patients are advised to avoid aerobic activities for several months after healing. Neonatal enterovirus infections can vary in how they respond to IVIG therapy. The majority of enterovirus infections are self‐limited, require no special therapy, and only little supportive care is required.

**Conclusion:**

The recent elevation in numbers for reported severe myocarditis in neonates within Wales and South West England, linked to enterovirus infection, poses a significant public health concern. Myocarditis caused by enteroviruses, particularly Group B coxsackieviruses, is associated with significant mortality rates. Diagnosis is supported by non‐invasive techniques and cardiac enzyme blood tests. Treatment modalities primarily involve a palliative approach.

## 1 INTRODUCTION

In the realm of public health, the World Health Organization (WHO) made a proclamation surmounting the constellation of occurrences, situated within the territories of Wales and South West England. On April 5, 2023, the National IHR focal point for the United Kingdom conveyed to the WHO an unusual upsurge of severe myocarditis, a perilous inflammatory cascade of the heart, in neonates intricately entwined with enterovirus infection.^1^ Enteroviruses belong to the lineage of the Picornaviridae family and are RNA‐based entities. As per the novel classification system based on genome sequencing techniques, enteroviruses are now subgroups into (HEV‐A), HEV‐B, HEV‐C, and HEV‐D, based on the resemblance in their viral structure protein gene.^2^ Neonates experience distinct and often more severe effects when exposed to enteroviruses compared to older children. As the pendulum of fear swings, the current situation in the region has brought attention and is being closely monitored by competent authorities.^3^

### 1.1 Epidemiology

Enteroviruses are one of the most prevalent types of virus that causes a huge burden of disease around the world. It is estimated that 10–15 million cases of symptomatic Enterovirus infections occur annually in the US alone. Children under the age of one are especially vulnerable, accounting for roughly 40% of Enterovirus infections. Nonpolio enterovirus is typically found in temperate places throughout the summer and early autumn, though outbreaks may continue into the winter in tropical settings circulation is year‐round or related to the rainy season.^4^

Neonatal enterovirus infections can occur before, during, or after birth. During the seasonal peaks, an unwell or febrile neonate should be evaluated in the community for enterovirus infection. Horizontal transmission can occur in a safe environment, such as a neonatal critical care unit (NICU), resulting in nosocomial outbreaks.^5^

Enterovirus infections present in a variety of ways. Asymptomatic infections account for around half of all cases. Symptomatic enterovirus infections range from nonspecific febrile symptoms to potentially fatal disorders like myocarditis and sepsis. Hand‐foot‐mouth illness, acute hemorrhagic conjunctivitis, and herpangina are the most common symptoms. In neonates with severe enterovirus infection, hepatitis is the commonest consequence but myocarditis has the highest mortality.^6^

### 1.2 Rise in enteroviral myocarditis

Enteroviruses are responsible for roughly 25%–35% of cases of confirmed myocarditis and pericarditis. Acute enterovirus myocarditis is most commonly caused by Group B coxsackieviruses. However, only a small percentage of enterovirus infections cause overt cardiac involvement.^4^

In recent years the UK has been seeing surges in the number of myocarditis cases in neonates caused by enterovirus. On April 5, 2023 WHO was informed about the rise in cases of neonatal severe myocarditis caused by enterovirus infection in Wales. Ten hospitalized neonates with a positive enterovirus polymerase chain reaction (PCR) test were reported to have myocarditis between June 2022 and April 2023. Seven of the 10 cases exhibited further subtyping, with coxsackie B3 or coxsackie B4 being detected. Patients presented with features of sepsis, myocarditis, or in cardiorespiratory arrest. One patient was still hospitalized as of May 5, 2023, and one had died.^1^

### 1.3 Diagnosis and management

All ages are affected by the deadly, frequently undiagnosed condition known as severe myocarditis, which has increased hospital admissions in the United Kingdom. Myocarditis has traditionally been diagnosed with an endomyocardial biopsy, in which samples are sent for histology, immunohistochemistry, and the PCR to look for probable infectious agents, most frequently enteroviruses. However, the most effective noninvasive diagnostic method at the moment is cardiovascular magnetic resonance imaging. There is a place for further diagnostic procedures, such as cardiac enzyme blood tests, ischemic electrocardiograms, and echocardiography, which look for left ventricular dysfunction in the presence of normal coronary arteries.^7^

Depending on the clinical manifestation and etiology, viral myocarditis is treated differently. If an underlying etiological agent is found, therapy is then focused on treating that factor. The majority of patients receive supportive care, which aims to manage any problems including heart failure or arrhythmias. Current European Society of Cardiology (ESC) recommendations for the treatment of acute heart failure apply. Emerging research supports the use of immunosuppressive medication in some circumstances. Patients are advised to avoid aerobic activities for several months after healing. Neonatal enterovirus infections can vary in how they respond to IVIG therapy. The majority of enterovirus infections are self‐limited, require no special therapy, and only little supportive care is required. However, because of the high mortality, it may be justified to administer intravenous immunoglobulin (IVIG) plus the antiviral medication pleconaril, if available, for life‐threatening infections, such as newborn infections, myocarditis, and disseminated infections.^8^ Extracorporeal membrane oxygenation can also be used in therapy for neonates with severe enteroviral myocarditis, as they may rapidly develop cardiovascular collapse, which may be resistant to standard medical treatment.^9^

### 1.4 Recommendations

There's no specific prevention for myocarditis. However, taking certain steps to prevent infections might help such as avoiding close contact with patients having symptoms of the flu or other respiratory illness until they have recovered. Physicians may be assisted in promptly detecting severe cases by being aware of the clinical symptoms, recognizing the risk factors and monitoring parameters linked with severe cases, and deploying techniques for prompt diagnosis such as a quick reverse‐transcription‐PCR for viral load. Furthermore, to give the fetus enough time to passively develop a protective antibody response, it is advised to delay delivery, if at all feasible, for at least 5–7 days following the onset of symptoms suggestive of maternal enterovirus infections.^10^

The WHO does not recommend any travel and trade restrictions to the United Kingdom based on the information available for this event.^1^ Currently, there is no vaccine against this, so control strategies during outbreaks concentrate on standard hygiene practices such as frequent handwashing and disinfecting dirty clothing and surfaces. To lessen the severity of the transmission, if the need arises it is advised to close daycare centers and schools. Additionally, continuous monitoring and surveillance methodologies are needed for better estimation of disease burden and to curb the spread to prevent the development of new cases.^11^, ^12^, ^13^

## 2 CONCLUSION

The recent elevation in numbers for reported severe myocarditis in neonates within Wales and southwest England, linked to enterovirus infection, poses a significant public health concern. Enteroviruses exhibit diverse clinical manifestations and are a major cause of the global disease burden, particularly affecting children under the age of one. Myocarditis caused by enteroviruses, particularly Group B coxsackieviruses, is associated with significant mortality rates. Diagnosis is supported by noninvasive techniques and cardiac enzyme blood tests. Treatment modalities primarily involve a palliative approach. Additional monitoring and research is required to better understand and address this public hazard.

## AUTHOR CONTRIBUTIONS

**Heeba Anis**: Writing—original draft; writing—review and editing. **Akbar B. Shaik**: Writing—original draft; writing—review and editing. **Angad Tiwari**: Visualization; writing—review and editing. **Abel Alemayehu**: Writing—original draft; writing—review and editing. **Abubakar Nazir**: Writing—original draft; writing—review and editing. **Linda Atulinda**: Writing—original draft; writing—review and editing. **Magda Wojtara**: Writing—original draft; writing—review and editing. **Olivier Uwishema**: Writing—original draft; writing—review and editing.

## CONFLICT OF INTEREST STATEMENT

The author declared no conflict of interest.

## ETHICS STATEMENT

The authors have nothing to report.

## TRANSPARENCY STATEMENT

The lead author Abubakar Nazir affirms that this manuscript is an honest, accurate, and transparent account of the study being reported; that no important aspects of the study have been omitted; and that any discrepancies from the study as planned (and, if relevant, registered) have been explained.

## Data Availability

The authors have nothing to report.
